# Episodes of Diversification and Isolation in Island Southeast Asian and Near Oceanian Male Lineages

**DOI:** 10.1093/molbev/msac045

**Published:** 2022-02-28

**Authors:** Monika Karmin, Rodrigo Flores, Lauri Saag, Georgi Hudjashov, Nicolas Brucato, Chelzie Crenna-Darusallam, Maximilian Larena, Phillip L Endicott, Mattias Jakobsson, J Stephen Lansing, Herawati Sudoyo, Matthew Leavesley, Mait Metspalu, François-Xavier Ricaut, Murray P Cox

**Affiliations:** 1 School of Natural Sciences, Massey University, Palmerston North, New Zealand; 2 Institute of Genomics, University of Tartu, Tartu, Estonia; 3 Institute of Computer Science, University of Tartu, Tartu, Estonia; 4 Laboratoire Evolution et Diversité Biologique (EDB UMR 5174), Université de Toulouse Midi-Pyrénées, CNRS, IRD, UPS, Toulouse, France; 5 Genome Diversity and Disease Laboratory, Eijkman Institute for Molecular Biology, Jakarta, Indonesia; 6 Department of Organismal Biology, University of Uppsala, Uppsala, Sweden; 7 Department Hommes Natures Societies, Musée de l’Homme, Paris, France; 8 Complexity Science Hub Vienna, Vienna, Austria; 9 Santa Fe Institute Center for Advanced Study in the Behavioral Sciences, Stanford University, Santa Fe, USA; 10 School of Humanities and Social Sciences, University of Papua New Guinea, National Capital District, Papua New Guinea; 11 CABAH and College of Arts, Society and Education, James Cook University, Cairns, QLD, Australia

**Keywords:** Y chromosome, human population genetics, Island Southeast Asia, phylogeography, migration

## Abstract

Island Southeast Asia (ISEA) and Oceania host one of the world’s richest assemblages of human phenotypic, linguistic, and cultural diversity. Despite this, the region’s male genetic lineages are globally among the last to remain unresolved. We compiled ∼9.7 Mb of Y chromosome (chrY) sequence from a diverse sample of over 380 men from this region, including 152 first reported here. The granularity of this data set allows us to fully resolve and date the regional chrY phylogeny. This new high-resolution tree confirms two main population bursts: multiple rapid diversifications following the region’s initial settlement ∼50 kya, and extensive expansions <6 kya. Notably, ∼40–25 kya the deep rooting local lineages of C-M130, M-P256, and S-B254 show almost no further branching events in ISEA, New Guinea, and Australia, matching a similar pause in diversification seen in maternal mitochondrial DNA lineages. The main local lineages start diversifying ∼25 kya, at the time of the last glacial maximum. This improved chrY topology highlights localized events with important historical implications, including pre-Holocene contact between Mainland and ISEA, potential interactions between Australia and the Papuan world, and a sustained period of diversification following the flooding of the ancient Sunda and Sahul continents as the insular landscape observed today formed. The high-resolution phylogeny of the chrY presented here thus enables a detailed exploration of past isolation, interaction, and change in one of the world’s least understood regions.

## Introduction

Island Southeast Asia (ISEA) and Near Oceania form a diverse interlocking geographical and cultural region with a population of over 400 million people. Comprising the Indonesia, Philippine, and Taiwan archipelagoes, ISEA hosts three of the five largest island states in the world, with Indonesia alone covering an area equivalent to that from Ireland to the Caspian Sea. Oceania is of course even larger, stretching from the eastern edge of ISEA, to New Guinea, and out to the furthest islands of the remote Pacific. Within Oceania, this study focuses on the Near Oceania region, particularly New Guinea and the Bismarck Archipelago, including their potential connections to Australia.

**Fig. 1.**
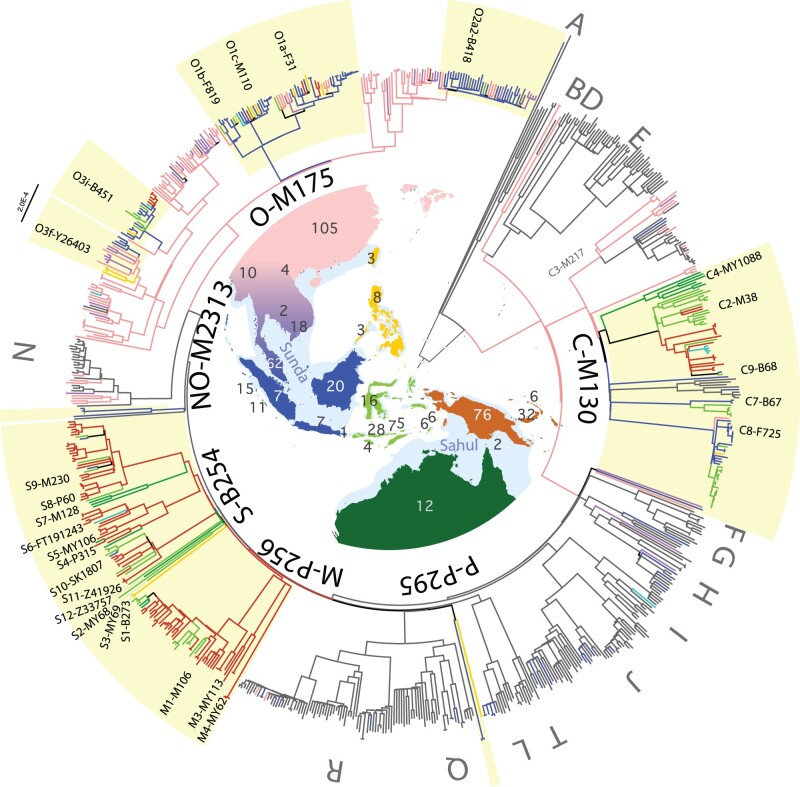
Paternal lineages from ISEA and Near Oceania in the context of the global chrY phylogeny. ML tree of 795 chrY sequences. Colors in the target region correspond to the geographic origin of the samples (numbers shown on the map): dark blue—western Indonesia and the Malay peninsula (Sunda); light green—Eastern Indonesia (Wallacean Islands), yellow—Philippines and Taiwan; brick orange—New Guinea and the Bismarck Archipelago (Northern Sahul); dark green—Australia (Southern Sahul); violet—Mainland Southeast Asia; pink—East Asia; bright blue—Pacific islands; gray—other world regions. The approximate maximum extent of the Sunda and Sahul land masses are shown on the map in light blue. Yellow boxes highlight lineages from the studied region. The root and the deepest splits have been shortened for better fit on the figure.

Over time, climatic changes have substantially transformed the physical landscape of this region from islands to continents and back. During the Pleistocene, long periods ∼65–15 thousand years ago (kya) saw many of today’s islands connected into substantial land masses, with the two largest called Sunda and Sahul (fig. 1) (Voris 2000). Deep sea trenches around Wallacean Islands kept these land masses separate (Hanebuth et al. 2000), but within them, lakes and river systems likely served as potential pathways for communication and movement, as they still do today. The Sunda and Sahul continents reached their maximum extent when sea levels were lowest during the last glacial maximum (LGM) ∼25 kya. Water levels subsequently rose with bursts of flooding taking place ∼14 kya, and the land links between modern islands and mainland Asia were largely drowned by ∼9 kya (Hanebuth et al. 2000; Voris 2000).

Archaeology shows that modern humans reached Australia ∼65–52 kya (O’Connell and Allen 2015; Clarkson et al. 2017), mainland New Guinea ∼49–40 kya (Summerhayes et al. 2010; Groube 1986), with the easternmost fringes of the Bismarck Archipelago being settled soon after, ∼45–35 kya (Leavesley and Chappell 2004; Torrence et al. 2004). Much later during the Holocene (<10 kya), the advent and widespread adoption of farming in mainland Asia triggered influential movements of people. This led to the spread of Austronesian languages and Neolithic practices through ISEA and out into Oceania, starting around 6 kya in Taiwan and reaching eastern Indonesia by ∼3.5 kya (Bellwood 2006; Xu et al. 2012; Kusuma et al. 2015; Brucato et al. 2016; Kirch 2017; Deng et al. 2020). Movements within the region, in part stimulated by independent instances of plant cultivation (Denham and Haberle 2008), were also extensive (Hudjashov et al. 2017). The result is that Indonesia and Papua New Guinea today host the richest linguistic diversity in the world, with over 700 and 800 languages spoken in those two modern nations. Coupled with this linguistic richness is an astonishing diversity of socio-cultural practices—post marital residence patterns, kinship systems, social norms, and community structures, many of which are only now transitioning away from traditional practices (Godelier 1982; Lansing et al. 2008; Guillot et al. 2013; Tumonggor et al. 2014).

Despite this extraordinary human diversity, knowledge of genetic diversity has lagged substantially behind other global regions. Of particular note is the Y chromosome (chrY), whose phylogeny has been resolved elsewhere in the world (Hallast et al. 2015; Karmin et al. 2015; Poznik et al. 2016), but is still only partially known in ISEA and Oceania (Mona et al. 2007; Karafet et al. 2010; Bergström et al. 2016). Despite the trend toward whole genome data (Hudjashov et al. 2017; Jacobs et al. 2019; Brucato et al. 2021; Larena et al. 2021), the lack of a robust regional chrY phylogeny has limited the identification and molecular dating of sex-specific historical processes. By undertaking extensive new regional sampling and chromosomal-level sequencing, this study presents the first fully resolved regional chrY phylogeny. Here, we fit newly identified lineages into the broader global phylogeny of chrY diversity and characterize patterns that reflect the specific regional history of ISEA and Near Oceania.

## Results and Discussion

### High-Resolution chrY Lineages for One of the Last Understudied Global Regions

To develop a fully resolved picture of chrY diversity in this region, we extracted chrY sequences from complete human genomes in a set of 152 samples sequenced on the Illumina platform that have not been analyzed before for their chrY diversity. This includes 14 newly reported samples (3 from New Guinea, 7 from Mentawai, and 4 from Sumba), 112 men from ISEA (Jacobs et al. 2019) and 26 men from Papua New Guinea (Brucato et al. 2021) (supplementary table S1, Supplementary Material online). Samples were chosen explicitly to fill gaps in earlier geographical coverage and lineage distributions. We then combined this new data set with previously published chrY sequences from the target region, as well as from broader geographical and phylogenetic contexts. The final global data set consisted of 795 full chrY sequences extracted from complete human genomes or from targeted sequencing, including samples from neighboring East Asia, Mainland Southeast Asia (MSEA), and other world regions (fig. 1 and supplementary table S1, Supplementary Material online).

### A Fully Resolved chrY Phylogeny with Robust Dates

Paternal lineages from ISEA and New Guinea typically fall into the major chrY haplogroups C-M130, M-P256, S-B254, and O-M175, respectively sampled here from 77, 50, 66, and 103 individuals (fig. 1 and supplementary table S1, Supplementary Material online). Distinct sublineages of C-M130 and O-M175 that occur in ISEA, New Guinea, and Australia largely fall within previously described diversity (Kayser et al. 2001; Karafet et al. 2010; Bergström et al. 2016; Nagle et al. 2016; Bergström et al. 2017). In contrast, M-P256 and S-B254 are typically restricted to New Guinea and neighboring islands and have much less well known phylogenies. We identify new phylogenetic structures and subhaplogroups, and resolve a substantial number of lineages that were previously classified only as paragroup K*-M526 (Kayser et al. 2006; Bergström et al. 2016; Nagle et al. 2016; Bergström et al. 2017). We also provide additional resolution to the rare lineage NO-M2313, previously classified as K2a1*-M2313 (Poznik et al. 2016). The result is a fully resolved phylogeny of regional chrY diversity, with all individuals uniquely assigned and no paragroups (fig. 1 and supplementary figs. S2–S4, Supplementary Material online). Sequence data allows dating of diversifications and expansions. Coalescence times were inferred using the Bayesian algorithms of BEAST (Drummond et al. 2012) and dates were calibrated using previously published split times and clock rates (supplementary table S2, Supplementary Material online) (Karmin et al. 2015).

Previous studies have highlighted the critical importance of robust filtering due to the complex repeat structure of the chrY (Karmin et al. 2015; Poznik et al. 2016). We employed a series of filters and sequence masks to limit the data set to high quality variant calls (detailed in Materials and Methods and supplementary table S9, Supplementary Material online), resulting in ∼9.7 Mb of complete chrY sequence per sample. Across the whole data set, there is an average of 648 (SD = 18) mutations from tips to the common BT node, with a unimodal and nonskewed distribution (supplementary fig. S1, Supplementary Material online). Each chrY lineage was assigned to a haplogroup according to the alternating letter/number rules of the Y Chromosome Consortium (Hammer 2002), taking account of additional modifications proposed subsequently to accommodate the volume of variant data from full chrY sequencing (Karmin et al. 2015). Because our study resolves several previously unresolved paragroups, we define a number of new clades in the global chrY tree, and necessarily relabel some previously known clades while keeping as close as possible to historic nomenclature.

### Major Radiations around 50 kya Reflect First Settlement in the Region

**Fig. 2.**
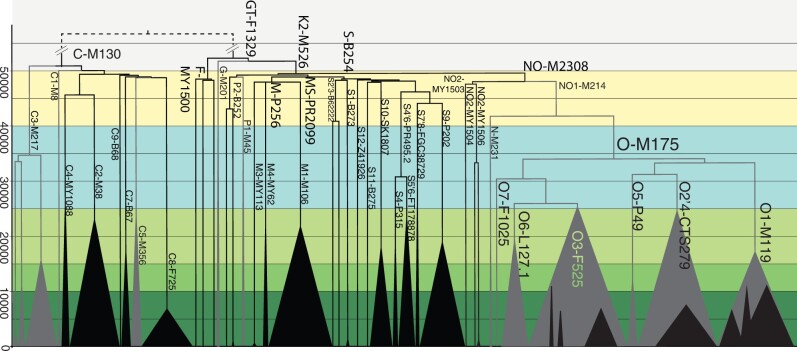
Region-specific lineages of C-M130, M-P256, and S-B254 diversified rapidly and early in contrast to later-arriving O-M175. Dated schematic Y chromosomal phylogenies of haplogroups C-M130 (*N* = 129), F-MY1500 (*N* = 3), M-P256 (*N* = 50), NO2-MY1503 (*N* = 5), O-M175 (*N* = 230), P-B252 (*N* = 3), and S-B254 (*N* = 66). Black—samples from ISEA and Near Oceania; gray—Mainland Asia and Eurasia. Lineages with coalescence dates more recent than ∼25 kya have been collapsed to emphasize the early branching events and the long pause ∼40–30 kya in many of the local lineages within haplogroups M-P256, S-B254, and C-M130, in contrast to the mainland Asian lineages of haplogroups O-M175 and C3-M217. The rare lineages of F-MY1500, P2-B252, and NO2-MY1503 all show early branching events, although their rarity prevents further conclusions about their later diversification history. Triangles are proportional to sample size, and background colors denote time windows as framed in the text. All full or target-captured chrY sequences available to us from ISEA, Oceania, and Australia are included. Haplogroups G-M201, P1-M45, and N-M231 are represented by only a few lineages; the main structures within C3-M217 and some O-M175 subgroups from other well-sampled global regions are represented, but thinned to a smaller number of individuals.

In concordance with previous findings from global chrY phylogenies (Karmin et al. 2015; Poznik et al. 2016), rapid diversification of lineages C-M130, M-P256, and S-B254 indicate human settlement of ISEA, New Guinea, and Australia over a very short time frame close to 50 kya. These major haplogroups arose in the region with few distinguishing mutations, indicating rapid diversification (fig. 2 and supplementary figs. S2–S4, Supplementary Material online). Even today, many of these branches have limited geographic distributions. Indeed, some remain rare single lineages with no further branching events, reflecting long-term isolation and small population size subsequent to this initial expansion event. Examples include S12-Z41926 in Australia, C9-B68, S2-MY68, and S3-MY69 in Indonesia, and M4-MY62 in New Britain (fig. 2 and supplementary figs. S2–S3, Supplementary Material online). In addition, rare lineages F-MY1500 (*N* = 3), P2-B525 (*N* = 3), and NO2-MY1533 (*N* = 5) present in MSEA and ISEA show a similar pattern of very early splitting events.

### C-M130, M-P256, and S-B254 Diversified Rapidly and Early

A small number of C-M130, M-P256, and S-B254 lineages from the early dispersal event dominate the eastern regions of ISEA and Near Oceania, whereas some are geographically widespread today. Within our extended data set, eight C-M130 subhaplogroups arose between 50 and 40 kya, of which five are found today in ISEA, New Guinea, and/or Australia (fig. 1 and supplementary fig. S2, Supplementary Material online). With 32 individuals carrying C2-M38, this is the lineage with the widest geographical distribution, reaching from the Wallacean Islands to as far east as Polynesia (fig. 1 and supplementary fig. S2, Supplementary Material online). Lineage C4-MY1088 has been sequenced in six Australian men and is restricted to Australia since its separation from C2-M38 ∼45 kya (95% CI: 41.6–49.6 kya) (supplementary fig. S2 and file S1, Supplementary Material online) (Bergström et al. 2016). Sequenced in 7 and 35 men, C7-B67 and C8-F725 stem from the early dispersal and became distributed more recently across both Sunda and the Wallacean Islands in modern western and eastern Indonesia, respectively (supplementary fig. S2, Supplementary Material online).

**Fig. 3.**
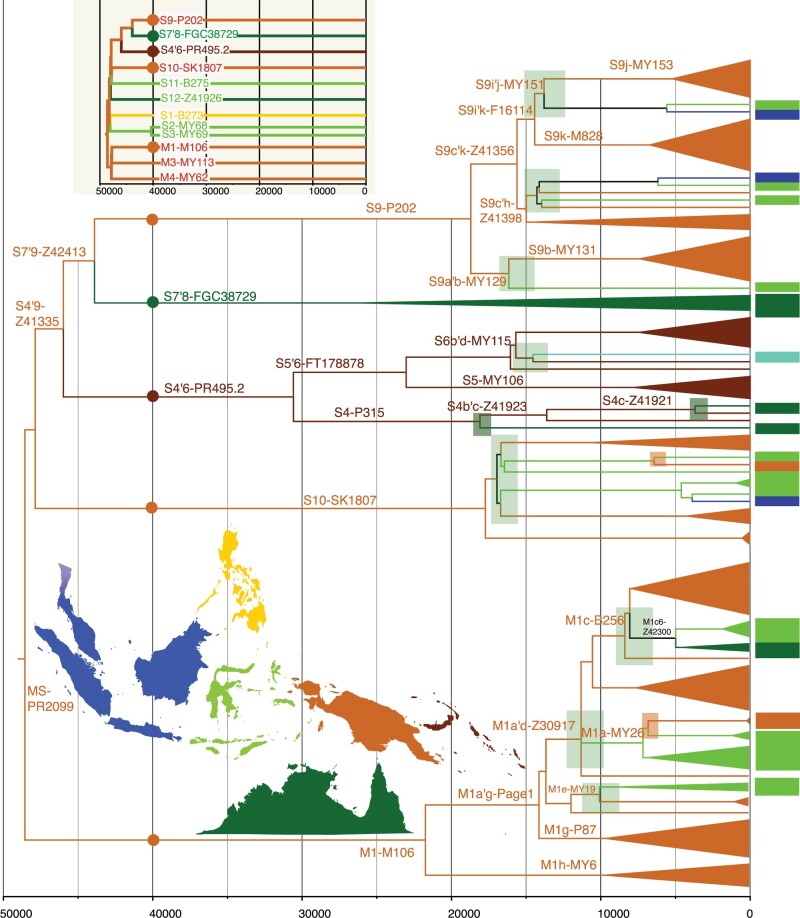
Contacts between Northern Sahul and neighboring areas within the MS-PR2099 lineages. The basic structure of haplogroup MS-PR2099 lineages is shown in the upper inset; circles denote sublineages that show evidence of contact between Northern Sahul and other regions, and are illustrated in more detail in the main figure. Colors on the tree and map denote the geographic origin of individuals. Light green boxes show likely westward paternal gene flow from Northern Sahul mostly to the Wallacean Islands, but also beyond; darker green boxes denote potential contact between New Britain and Australia. Light brick brown boxes show likely back migration to New Guinea from Wallacea. Light teal represents lineages from Pacific islands. For more details, see supplementary figure S3 and tables S1 and S4, Supplementary Material online.

Before splitting ∼49 kya (95% CI: 45.9–51.3 kya), haplogroups M-P256 and S-B254 (fig. 2 and supplementary fig. S3, Supplementary Material online) shared five common mutations (supplementary table S7, Supplementary Material online). Subsequently, both lineages accumulated only a few haplogroup-defining variants, instead rapidly diversifying further. Before ∼40 kya, M-P256 split into three lineages and S-B254 into eight (fig. 2 and supplementary fig. S3, Supplementary Material online). All lineages that we have sampled from this early diversification further split much later, some even after tens of thousands of years. Today, all sampled M-P256 lineages are present in New Guinea and the Bismarck Archipelago, with two lineages restricted to that area. The most numerous lineage, M1-M106 with 46 individuals, also has the widest geographic distribution, with individuals from the Wallacean Islands of eastern Indonesia and the Torres Strait nested within New Guinean diversity (fig. 3 and supplementary fig. S3 and file S2, Supplementary Material online).

Many of the eight haplogroup S-B254 lineages (fig. 2, inset of fig. 3 and supplementary fig. S3, file S2, and table S4, Supplementary Material online), which also arose during this same time period of 50–40 kya, are geographically restricted. Of these, four Australian individuals belong to S7′8-FGC38729 and one to S12-Z41926, whereas S1-B273 is found in two Philippine men, but S9-P202 and S10-SK1807 are spread widely across eastern Indonesia and New Guinea with 31 and 16 individuals, respectively (fig. 3 and supplementary fig. S3, Supplementary Material online). S4′6-PR495.2, present in 16 men, is mostly specific to New Britain and first split around 30 kya (95% CI: 28.0–33.2 kya), well after the time of first settlement of New Britain (Pavlides and Gosden 1994; Leavesley and Chappell 2004), and today has sublineages distributed as far as Australia and Polynesia. Strictly Australian lineages, such as S7′8-FGC38729, diverged around this time, with evidence of only limited later contact (supplementary fig. S3, Supplementary Material online). The overall picture ∼50–40 kya is therefore one of extensive early population movements across the region associated with rapid lineage radiation, in agreement with archaeological evidence showing that early settlers spread quickly across Sahul within a few thousand years (Bowler 2003; Leavesley and Chappell 2004; Summerhayes et al. 2010; Clarkson et al. 2017).

### A Long Pause Followed Initial Settlement

Regional chrY lineages show a striking lack of new diversity between 40 and 25 kya (fig. 2 and supplementary figs. S2 and S3 and tables S3 and S4, Supplementary Material online). Existing lineages in C-M130, M-P256, and S-B254 survived through this gap, but most show no evidence of further radiation during this time. This striking lack of new diversity between 40 and 25 kya is also observed in the regional mitochondrial DNA (mtDNA) lineages (Pedro et al. 2020). ChrY haplogroup S4′6-PR495.2, which is mostly found in New Britain (eastern Papua New Guinea), is the only lineage that split during the hiatus, ∼30 kya (95% CI: 28.0–33.2 kya). Noting the limitation that modern sampling may miss lineages that have gone extinct, the observed widespread pause in phylogenetic diversification appears to be characteristic of low population size and persistent population structure during much of the Upper Pleistocene, in agreement with maternal lineage patterns (Pedro et al. 2020), the relatively small numbers of Late Pleistocene archaeological sites identified (Williams 2013; Summerhayes et al. 2017) and the strong territoriality and geographical stability seen in modern ethnographic studies of New Guinea and Indigenous Australian societies (Muke 1993; Sutton 2003). In notable contrast, haplogroup O-M175 on Mainland Asia, which is now the dominating lineage in Eastern Eurasians and comprises more than a quarter of all male lineages in the world (Yan et al. 2014), continued to radiate throughout the Pleistocene. About 33 kya (95% CI: 30.6–36.6 kya), O-M175 began to diversify rapidly, and during the next 10,000 years, six major lineages arose (fig. 2 and supplementary fig. S4, Supplementary Material online), which are now spread across Asian mainland. Some of these sublineages later swept into ISEA and out into the Pacific (Kayser et al. 2006; Karafet et al. 2010). Therefore a distinction exists between Mainland Asia, with extensive population expansions and mobility throughout the Upper Pleistocene, and ISEA, New Guinea with Australia, where phylogenies reflect much smaller population sizes and more restricted male mobility, which may have been enhanced both by geography and local cultural practices.

### A Second Bout of Diversification 25–15 kya Included a Split between New Guinean and Australian Lineages

After 25 kya, major periods of lineage radiation are observed across ISEA, New Guinea, and Australia. These lineages stem from different chrY subbranches arising from northern Sahul diversity (fig. 3 and supplementary fig. S3, Supplementary Material online) and they dispersed at different times. The most numerous Australian lineage in this sample, C4-M347 (carried by six men), diversified between 25 and 17 kya, coinciding with a major split ∼26 kya (95% CI: 23.2–28.9 kya) in a second Australian lineage S7′8-FGC38729 present in three men (supplementary figs. S2 and S3, Supplementary Material online). About 23 kya (95% CI: 20.3–25.5 kya), C2-M38 diverged into two lineages, one now found from Wallacea (eastern Indonesia) to New Britain, with the other largely radiating within mainland New Guinea (supplementary fig. S2, Supplementary Material online). M1-M106 split ∼21 kya (95% CI: 19.5–23.9 kya) into M1a′g-Page1 and M1h-MY6 (42 and 4 men, respectively), and is found today in New Guinea and New Britain, but also radiated westward (fig. 3 and supplementary fig. S3, Supplementary Material online). The most numerous sublineage in New Guinea, S9-P202, split ∼19 kya (95% CI: 17.1–20.5 kya), with subsequent expansion ∼15–13 kya creating off-shoots of different sublineages across the Wallacean Islands of Alor, Lembata, Sulawesi, and Borneo (fig. 3 and supplementary fig. S3, Supplementary Material online). Over the same timeframe, C7-B67 split ∼17 kya (95% CI: 14.6–19.6 kya) and spread between Borneo, Flores, and Lembata (supplementary fig. S2, Supplementary Material online), and S10-SK1807 gave rise to descendant lineages in New Guinea, New Ireland, and Eastern Indonesia around the same time (95% CI: 15.9–19.6 kya) (fig. 3 and supplementary fig. S3, Supplementary Material online). Lineages with New Guinean origins are mostly found on the Wallacean Islands, with a few in other regions as well.

The prevailing view of regional history is of two major human movements—the initial settlement (Leavesley and Chappell 2004; Summerhayes et al. 2010; Clarkson et al. 2017; O’Connell et al. 2018) and the arrival of Austronesian speakers 3–3.5 kya (Bellwood 2004, 2006), implicitly suggesting that little occurred in this region during the Upper Pleistocene. However, the more resolved chrY tree with denser sampling uncovers a far more complex paternal demographic history, consistent with some of the newer studies in genetics (Gomes et al. 2015; Pedro et al. 2020; Purnomo et al. 2021), linguistics (Schapper 2017), and archaeology (Summerhayes 2007; Summerhayes et al. 2017; Bellwood 2019). In particular, the increase in genetic lineage diversification correlates well with increasing population interactions and population sizes postulated from the archaeological record around 25–20 kya, based on animal, plant, and object (e.g., obsidian) translocation between Northern Sahul regions, changes in the subsistence economy, increasing occupation of Highland New Guinea, and settlement of distant islands (e.g., Manus), all of which suggest population size increases and connection of local dynamics to larger regional networks (Leavesley and Chappell 2004; Specht 2005; Summerhayes 2007; Summerhayes et al. 2017).

We see these connections within a number of haplogroups. In S-B254, the first split within the S4′6-PR495.2 lineages of New Britain occurred earlier, ∼30 kya (95% CI: 28.0–33.2 kya). Next, S5′6-FT17887 diversified first ∼23 kya (95% CI: 20.9–25.3 kya) and then ∼16 kya (95% CI: 14.4–17.7 kya) (fig. 3 and supplementary fig. S3, Supplementary Material online). Its sister lineage, S4-P315, appears to have been isolated for some time, only diverging ∼18 kya (95% CI: 15.9–20.3 kya) when it split between the New Britain and Queensland (Australia) groups. A much more recent split ∼3.6 kya (95% CI: 2.7–4.7 kya) is also seen between men from these two locations (fig. 3 and supplementary fig. S3, Supplementary Material online). Hence, despite perceptions of long-term isolation between New Guinea and Australia (Bergström et al. 2016; Nagle et al. 2016), joint analyses of chrY from Papua New Guinea (Vernot et al. 2016) and Australia (Bergström et al. 2016) reveals phylogenetic relationships between lineages from New Britain and Australia that are much older than previously thought. This phylogenetic connection is beyond simply the Torres Strait region and is similar to that seen in autosomal data (Brucato et al. 2021). The coalescence date suggests an upper estimate of the Late Pleistocene for possible contact between these regions (fig. 3 and supplementary fig. S2, Supplementary Material online).

Although no archaeological evidence supports direct contact between New Britain and Australia, the Torres Strait Islands (between New Guinea and Australia) may have served as a bridge between these two regions. Indeed, trading and intermarriage has been reported between Torres Strait Islanders and Indigenous Australians (Beckett 1987), and some New Guinean lineages (mtDNA Q1, P2 and P3, and chrY M1c6-Z42300) are shared by men with Torres Strait ancestry (supplementary fig. S3, Supplementary Material online) (Nagle et al. 2016; Pedro et al. 2020). This genetic affinity between Bismarck Islanders and Indigenous Australians may also result from genetic exchanges predating the divergence between these populations during the postglacial period (18–10 kya) (fig. 3 and supplementary fig. S3, Supplementary Material online) (Nagle et al. 2016; Pedro et al. 2020; Brucato et al. 2021). More recent contact was also possible during the rapid spread of the Lapita Cultural Complex, which originated in the Bismarck Archipelago 3.3 kya (Denham et al. 2012), spread to the south Papuan Coast 2.9 kya (David et al. 2019), and may have subsequently influenced islands off the Queensland coast by 2.5 kya (Lilley 2019).

**Fig. 4.**
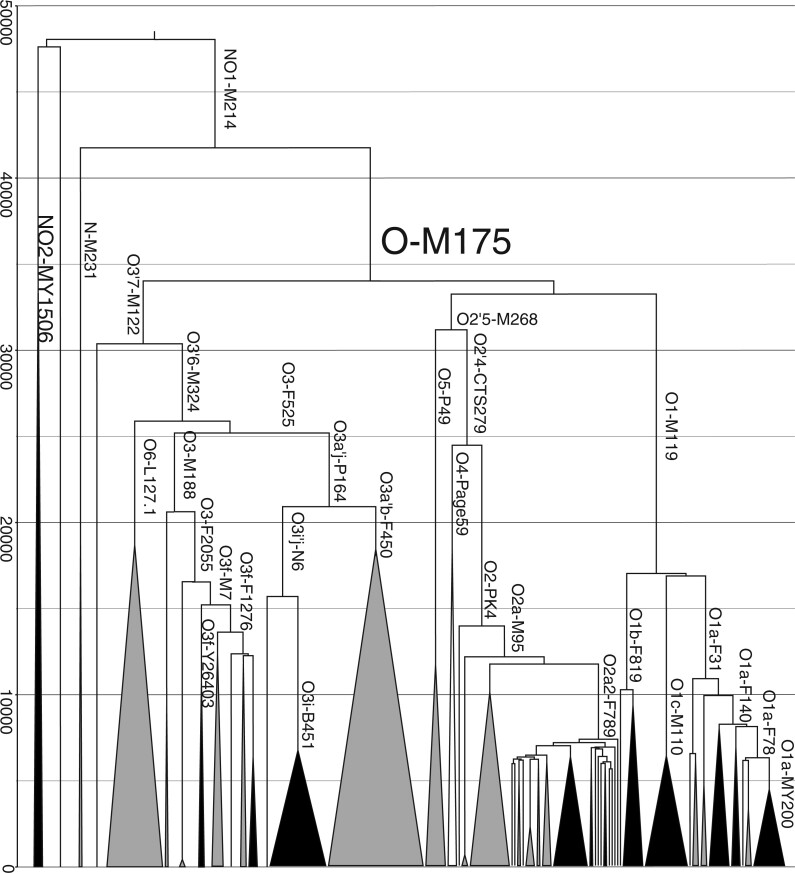
Haplogroup O-M175 sublineages in ISEA, New Guinea, and Oceania. A number of distinct sublineages (black) of O-M175 have penetrated from the Asian mainland into ISEA and beyond, and are likely associated with the expansion of Austronesian speakers. Most of these lineages coalesce ∼6–5 kya within subsets of the wider Asian diversity, except for O1b-F819 and O3f-M7 sublineages, which have earlier diversification times (details in supplementary fig. S4 and table S1 and S5, Supplementary Material online). In contrast, lineage NO2-MY1506, found today on Sunda and Sulawesi, split very early from its South Asian sister lineage.

Also during this time period (25–15 kya), the sublineages of haplogroup O-M175 continued to radiate (fig. 4 and supplementary fig. S4, Supplementary Material online). Sublineages of O3-F525, today widely distributed on Mainland Asia and ISEA split ∼25 kya (95% CI: 18.9–28.3 kya), including the formation of the major branch O3a′j-P164, which includes the later expansion of the two most numerous paternal lineages found in Han Chinese (Yan et al. 2014). Between ∼18 and 15 kya, expansions and splits occurred within several lineages in parallel, notably O1-M119, O4-Page59, O6-L127.1, O3a′b-F450, O3f′h-M2055, and O3i′j-N6 (fig. 4 and supplementary fig. S4 and table S5, Supplementary Material online).

### An Explosion of Activity Occurred at the Pleistocene-Holocene Boundary 15–10 kya

The sea level began rising ∼14 kya with flooding bursts over the next five millennia leading to the current island landscape (Hanebuth et al. 2000; Voris 2000). During this time of major geologic changes, the autosomal genetic data indicates a marked increase of expansion and diversification in the Philippines (Larena et al. 2021), gene flows between islands in the region (Brucato et al. 2021), and the maternal mtDNA data from Sahul and Wallacea shows population expansion (Pedro et al. 2020). Similarly, archaeological data suggest a strong population increase from the Holocene transition onwards, as part of a longer process that started in the late Pleistocene (25–20 kya) and culminated with the Austronesian dispersal across the Northern Sahul region (Specht 2005; Bellwood 2006; Summerhayes 2007; Donohue and Denham 2010; Williams 2013; Summerhayes et al. 2017). We also see a marked increase in the radiation of chrY lineages, coupled with extensive mobility across the region. Haplogroup S-B254 shows splits in New Britain between S4b-MY103 and S4c-Z41921, with the lineage later shared between men from New Britain and Queensland (fig. 3 and supplementary fig. S3, Supplementary Material online). Haplogroup M-P256 lineages in New Guinea and New Britain also diversified during this time. Between ∼14 and 9 kya, the most numerous and widely spread subbranch M1a′g-Page1 expanded rapidly within New Guinea (M1b-MY39, M1c-B256, M1e′f-Z43413) and New Britain (M1g-P87), with some lineages spreading west to eastern Indonesia (M1e-MY19, M1a-MY26) (fig. 3 and supplementary fig. S3, Supplementary Material online). Within haplogroup C-M130, ∼13–10 kya, a series of divergences within C2a-M208 in New Guinea gave first an offshoot to Wallacea and then formed the lineages that, much later, spread out into the Pacific (supplementary fig. S2, Supplementary Material online) (Cox et al. 2007). Within the few Australian lineages, a split in C4-MY1088 in Queensland occurred ∼11 kya (95% CI: 9.2–13.0 kya) (supplementary fig. S2, Supplementary Material online).

During this time, haplogroup O-M175 diversification patterns are similar to those of other chrY haplogroups in the region. Between 14 and 10 kya, major splits occurred in O1a-F31, O2-PK4, and many other O-M175 lineages, some of which are shared between Mainland and ISEA. Of particular note, O3f-M7 diversified ∼15 kya (95% CI: 11.2–17.4 kya), giving rise to branches found on Mainland Asia, Taiwan, and the Philippines, with the latter two lineages diverging from each other ∼10 kya (95% CI: 7.6–13.0 kya) within O3f-Y26403. O3f-M7 occurs widely across western Indonesia at low frequency today, and had previously been thought to reflect connections with mainland China during the historic era (Karafet et al. 2010). Although sampling remains low, the current O3f-M7 phylogeny suggests a potentially much earlier upper limit to the movement of people carrying this lineage, either in the Late Pleistocene or starting ∼15 kya (fig. 3 and supplementary fig. S4 and table S5, Supplementary Material online). Older connections between Mainland Asia and western Indonesia were also recently shown by high levels of pre-Neolithic East Asian ancestry in autosomal genetic data of the young female buried ∼7.3 kya at Leang Panninge cave in Sulawesi (Carlhoff et al. 2021).

### Holocene Dispersals Carried Few External Lineages from Mainland East Asia

The sea had risen to near its current level by 8–7 kya, leading to the creation of rich coastal floodplains (Terrell et al. 2011), and rising temperatures gradually led to the higher altitudes of New Guinea being more suitable for permanent human occupation (Summerhayes et al. 2017). Indigenous food plant cultivation in the highlands of New Guinea began ∼10 kya (Hope and Haberle 2005; Denham and Haberle 2008), coinciding with the expansion seen in M1-Page1 and various S-B254 sublineages (fig. 3 and supplementary table S4, Supplementary Material online). However, the Holocene story is in many ways dominated by haplogroup O-M175. From 10 to 2 kya, many expansion events occurred within haplogroup O-M175 (fig. 4 and supplementary fig. S4, Supplementary Material online), including the rise of the most numerous paternal lineages in the world today (Yan et al. 2014). Within ISEA, O3i-B451 and O2a2-F789 stem from this time of major haplogroup O-M175 expansion. However, the lineage with the greatest impact was O1-M119, which arose ∼33 kya (95% CI: 24.5–36.9 kya), diverged into sublineages ∼17 kya (95% CI: 12.3–19.3 kya), but underwent a striking period of radiation during the mid-Holocene (fig. 4 and supplementary fig. S4 and table S5, Supplementary Material online).

O1-M119 contains three main sublineages: O1a-F31, found in 35 men from across Mainland Asia, ISEA, and in New Guinea; O1b-F819 found in nine men from the Philippines, Sunda, and the Wallacean islands; and O1c-M110 found in 16 men from the Philippines, western Indonesia, and New Guinea. O1a-F31 radiated rapidly between ∼8 and 4 kya, with a subbranch in Taiwan and Malaysia separating ∼4 kya (95% CI: 2.7–4.9 kya). O1b-F819 lineages in the Philippines and Indonesia split ∼9 kya (95% CI: 7.0–11.0 kya), but radiated rapidly ∼4 kya (95% CI: 3.1–5.0 kya). Today, O1c-M110 is found only within ISEA and New Guinea, where it shows evidence of rapid expansion 6–5 kya (fig. 4 and supplementary fig. S4 and table S5, Supplementary Material online). We note the limitation that all molecular dates have some level of uncertainty (supplementary tables S3–S5, Supplementary Material online), and not all known O1-M119 diversity is included here (Sun et al. 2021). Nevertheless, the striking radiations observed are characteristic of a diverse population of Mainland Asian origin moving into ISEA and expanding rapidly. Due to their timing and current geographical distribution, these genetic lineages are typically associated with Austronesian-speaking peoples and Neolithic practices, which spread over a period of time, starting around 6 kya in Taiwan and reaching eastern Indonesia and New Guinea ∼3.5 kya, before subsequently moving out into Oceania (Bellwood 2006; Karafet et al. 2010; Xu et al. 2012; Kirch 2017; Deng et al. 2020).

### Holocene Expansions in Indigenous chrY Lineages

Although arrivals from Mainland Asia have been the traditional focus of mid-Holocene studies in ISEA, we identify a series of simultaneous radiations in lineages with long-standing local connections in C-M130, M-P256, and S-B254. In addition, C8-F725 seeded many new lineages in a short time period between ∼6.5 and 5 kya, including radiating into the Philippines, western Indonesia (Borneo and Mentawai), and the Wallacean islands of eastern Indonesia (supplementary fig. S2, Supplementary Material online). A second wave of expansions occurred around ∼4–3 kya when lineages in Borneo split from their neighbors in Malaysia or Sulawesi. Local interisland radiations on Flores and Mentawai also began ∼2.5–1.5 kya (supplementary fig. S2, Supplementary Material online). In haplogroup M1a′g-Page1, multiple lineages experienced radiations between 7.5 and 2 kya (downstream of M1c-B256, M1b-MY39, M1a-MY26, and M1g-P87), mostly within New Guinea, Wallacea, and the Torres Islands (fig. 3 and supplementary fig. S3, Supplementary Material online). In haplogroup S-B254, the previously mentioned split within S4c-Z41912 also appeared between Queensland (Australia) and New Britain ∼3.6 kya (95% CI: 2,730–4,719 kya) (fig. 3 and supplementary fig. S3, Supplementary Material online). Many of these expansions and population movements that occurred after ∼4 kya were likely triggered by the introduction of agriculture from Austronesian-speaking cultural communities (Deng et al. 2020; Alam et al. 2021), perhaps coupled in some places with the growing influence of local Papuan agricultural practices that had commenced as early as ∼10 kya (Denham and Haberle 2008).

### Population Mobility and Lineage Expansion Has Continued to the Present

Of course, chrY lineage evolution continues, with local expansions observed across nearby islands within the last two millennia. On Flores, sublineages that shared a common ancestor as far back as 49 kya expanded rapidly and locally within the past few thousand years (such as C8-F725, C7-B67, and M1c6-Z42300). Similar patterns of recent expansion are seen in western Indonesia (C8-F725, O3i-B450), eastern Indonesia (M1a3-MY33, O3i-B452), and New Guinea (C2a-M208), including the concomitant expansion of this same C2 lineage into and within Polynesia. Many of the fundamental driving forces observed during the earlier periods of ISEA, New Guinea, and Australian history have therefore continued right up to the present.

## Conclusions

Phylogenetic relationships of the major paternal genetic lineages of ISEA and Near Oceania are among the last in the world to be resolved. Analyzing ∼9.7 Mb of chrY sequence from a geographically and culturally diverse set of men has enabled us to resolve and date the phylogeny from this region. Two well-known population expansions are confirmed, with multiple rapid diversifications between ∼50 and 40 kya reflecting rapid early settlement, and lineages expanding ∼6–5 kya indicating extensive movements and interactions. Interestingly, in the period ∼40–25 kya, between these major bursts of chrY diversity, we observe almost no branching events, with old lineages persisting but not diversifying. A similar pause in diversification is apparent in maternal mtDNA lineages from the region (Pedro et al. 2020). Starting from the LGM and intensifying in the subsequent warming period, multiple diversification events follow the flooding of the Sunda and Sahul continents as the insular landscape we see today formed (Voris 2000) and local populations dispersed.

The improved chrY topology also highlights more localized events with important historical implications. O3-M7 lineages from Taiwan and the Philippines diverged from mainland Asian groups ∼15 kya, earlier than previously thought (Karafet et al. 2010). This coalescence date provides an upper limit to the movements of people, possibly supporting the growing view from other evidence of early movements between MSEA and ISEA well predating the Neolithic period (Karafet et al. 2010; Trejaut et al. 2014; Vallée, Luciani and Cox 2016). Despite the scarcity of chrY sequence data from Australia (Bergström et al. 2016), we find that some Australian lineages are nested within New Britain diversity, indicating that Australia experienced at least some interaction with the Papuan world. These and other contacts emphasize the potential of less common chrY lineages to illuminate the subtleties of regional contacts. The resolved phylogenetic framework of the chrY presented here will thus enable future exploration of isolation, interaction, and change in one of the world’s least understood regions.

## Materials and Methods

### Human Subjects

To develop a fully resolved picture of chrY diversity in this region, we sequenced 14 new male samples (fig. 1 and supplementary table S1, Supplementary Material online) on the Illumina platform, and combined these with 26 chrY sequences from Papua New Guinea (Brucato et al. 2021) and 112 chrY sequences from ISEA (Jacobs et al. 2019) that were not previously analyzed for their chrY diversity. We then combined this new data set with all available chrY sequences from the region, supplemented with samples from a broader geographic and phylogenetic context taken from previously published full chrY studies on neighboring East Asia, Mainland Southeast Asia, and other world regions (Abecasis et al. 2010; Drmanac et al. 2010; Wong et al.2013; Carmi et al. 2014; Ball et al. 2014; Karmin et al. 2015; Bergström et al. 2016; Ilumäe et al. 2016; Malaspinas et al. 2016; Mallick et al. 2016; Poznik et al. 2016; Vernot et al. 2016; Zheng-Bradley et al. 2017; Hudjashov et al. 2018; Bergström et al. 2020; Byrska-Bishop 2021). The final data set comprised 795 samples (supplementary table S1, Supplementary Material online).

### Ethics

The donors of all samples provided written informed consent and all experiments were performed in accordance with the relevant guidelines and regulations of collaborating institutions. Collections in Indonesia followed protocols for the protection of human subjects established by the Eijkman Institute Research Ethics Commission (EIREC#90), Nanyang Technological University (IRB-2014-12-011) and the University of Arizona. Permission to conduct research in Indonesia was granted by the State Ministry of Research and Technology (RISTEK). Collections in Papua New Guinea were approved by the Medical Research Advisory Committee of Papua New Guinea (National Department of Health) under research ethics clearance MRAC 16.21 and by the French Ethics Committees (Committees of Protection of Persons CPP 25/21_3, n°SI : 21.01.21.42754). Permission to conduct research in Papua New Guinea was granted by the National Research Institute of Papua New Guinea (permit 99902292358).

### Data Availability

The complete chrY sequences analyzed here for the first time have been deposited in the European Genome-Phenome Archive under accession numbers EGAS00001006028 and EGAS00001006025.

### Sequencing, Mapping, and Genotyping

All newly generated chrY sequences were generated using the Illumina technology (Illumina, San Diego, CA, USA) on the HiSeq instrument (PCR-free protocol) with 30× genome-wide coverage. We employed a series of filters and sequence masks to limit the data set to high quality variant calls, resulting in 9.7 Mb of complete chrY sequence per sample. We used the same processing pipeline for all Illumina data. FASTQ files were mapped with BWA-MEM v0.7.12 (Li and Durbin 2009) to the human reference hs37d5 (http://ftp.1000genomes.ebi.ac.uk/vol1/ftp/technical/reference/phase2_reference_assembly_sequence; last accessed March 8, 2022). Read duplicates were removed with Picard v2.12.0 (http://broadinstitute.github.io/picard/; last accessed March 8, 2022) and the remaining unique reads were realigned around known indels, followed by base quality score recalibration (BQSR) using GATK v3.8 (Poplin et al. 2018). Variant calling was performed with GATK HaplotypeCaller in haploid mode. All-sites VCF files were filtered with bcftools v1.9 (Li 2011). The Illumina data and previously filtered data from the Complete Genomics technology (supplementary table S1, Supplementary Material online) were merged with GATK CombineVariants v3.8 (Poplin et al. 2018). We extracted the effective overlap between the two data sets by masking out all positions with a 5% or higher proportion of missing genotypes in either the Illumina or Complete Genomics data sets. We additionally excluded regions with poor mappability as described previously (Karmin et al. 2015; Poznik et al. 2016), resulting in a total of 9.7 Mb of analyzed chrY sequence. Variant positions used for phylogenetic reconstruction in each haplogroup are listed in supplementary tables S6–S8, Supplementary Material online.

### Phylogeny Reconstruction and Dating

For the complete data set of 795 samples, a maximum-likelihood (ML) tree was constructed with GTRCAT using 200 rapid bootstrap inferences followed by a thorough ML search executed by RaXML v8.0.0 (Stamatakis 2014) (fig. 1 and supplementary fig. S1, Supplementary Material online). Initial haplogroup labels were assigned with yHaplo (Poznik 2016). All identified variants for shared nodes were annotated to the ML trees and curated manually (supplementary tables S6–S8, Supplementary Material online). We then used the software package BEAST v1.7.5 (Drummond et al. 2012) to simultaneously reconstruct phylogenies and estimate coalescence times. We ran three separate lineage-based analyses for haplogroups C-M130, MS-PR2099, and O-M175. For each haplogroup we used the same parameters: a Bayesian skyline coalescent tree prior, the general time reversible (GTR) substitution model with gamma-distributed rates, a relaxed lognormal clock and the piecewise-constant coalescent model with the number of groups set to 10. For each haplogroup, four parallel runs with different random number seeds were performed. Each run had at least 60 million chains logged every 3,000 steps. The results were visualized and checked for effective sample size above 200 using Tracer v1.4. Four parallel runs were combined with LogCombiner and the initial 25% discarded as burn-in. Coalescence time estimates were computed with normally distributed age priors from a previously published phylogeny that had used a mutation rate of 0.74 × 10^−9^ (95% CI: 0.63–0.95 × 10^−9^) per base per year (Karmin et al. 2015). The standard deviation (SD) was set to cover the published confidence intervals of the calibration nodes (supplementary table S2, Supplementary Material online). For haplogroups MS-PR2099 and O-M175, we used an extended set of samples to provide the broader global structure of the phylogeny and the same two samples from haplogroup C-M130 as the outgroup (supplementary figs. S2 and S4, Supplementary Material online). The BEAST analyses of MS-PR2099 had 154 individuals and 17,621 variants (supplementary fig. S3 and table S7, Supplementary Material online), whereas O-M175 had 259 individuals and 19,743 variants (supplementary fig. S4 and table S8, Supplementary Material online). For both phylogenies, the calibration point for coalescence age estimates was set for the GT-F1329 node with an age of 51,557 years (95% confidence interval [CI]: 50,206–52,917) (Karmin et al. 2015) and a SD of ±1,355 in the BEAST analyses. The phylogeny of haplogroup C-M130 was rooted with two samples from J-M304 (supplementary fig. S2, Supplementary Material online) and consisted of 131 individuals with 9,977 variant positions (supplementary table S6, Supplementary Material online). The calibration point was set to the root of haplogroup C-M130 with an age of 50,865 years (95% CI: 49,191–52,699) (Karmin et al. 2015), with SD set to ±1,835 in BEAST. We used Newick Utilities (Junier and Zdobnov 2010) for tree processing, statistical analyses and plotting was conducted in R v4.1.2 (R Core Team, 2014) with the package ggplot2 (Wickham, 2009). All trees were visualized using FigTree v1.4.4 (http://tree.bio.ed.ac.uk/software/figtree/; last accessed March 8, 2022).

## Supplementary Material

Supplementary data are available at *Molecular Biology and Evolution* online.

## Supplementary Material

msac045_Supplementary_Data
