# Supplementary material for: Episodes of Diversification and Isolation in Island Southeast Asian and Near Oceanian Male Lineages
Source: Mol Biol Evol. 2022 Feb 28;39(3):msac045. doi: 10.1093/molbev/msac045 (PMC8926390; doi:10.1093/molbev/msac045)
Supplement: msac045_Supplementary_Data [file msac045_supplementary_data.zip › MBE-21-1195_Karmin_Supplemental_Figures_S1-S4.pdf]

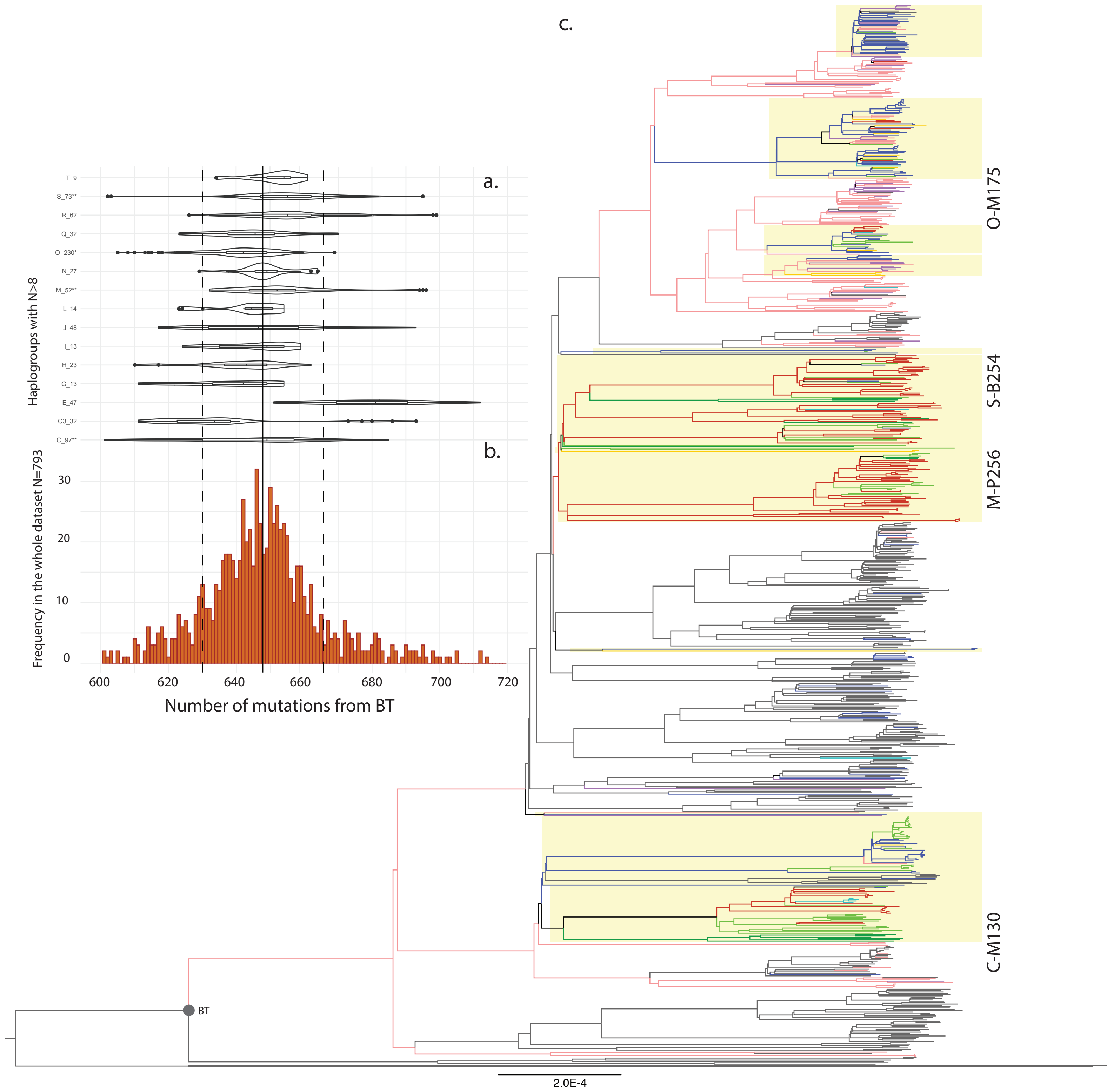

Figure S1 Distribution of the number of mutations from terminal tips to the BT node (gray circle) on the Maximum Likelihood (ML) tree.
a. violin plots for haplgroups (hgs) with N>8, number of samples is next to the hg label, main hgs discussed in the text are marked with **.
b. histogram for the whole dataset of 793 individuals with mean (648) and standard deviation (18).
c. the ML tree as in Figure 1, yellow boxes highlight the main hgs discussed in text.
The two rooting samples from hg A are excluded from calculations.

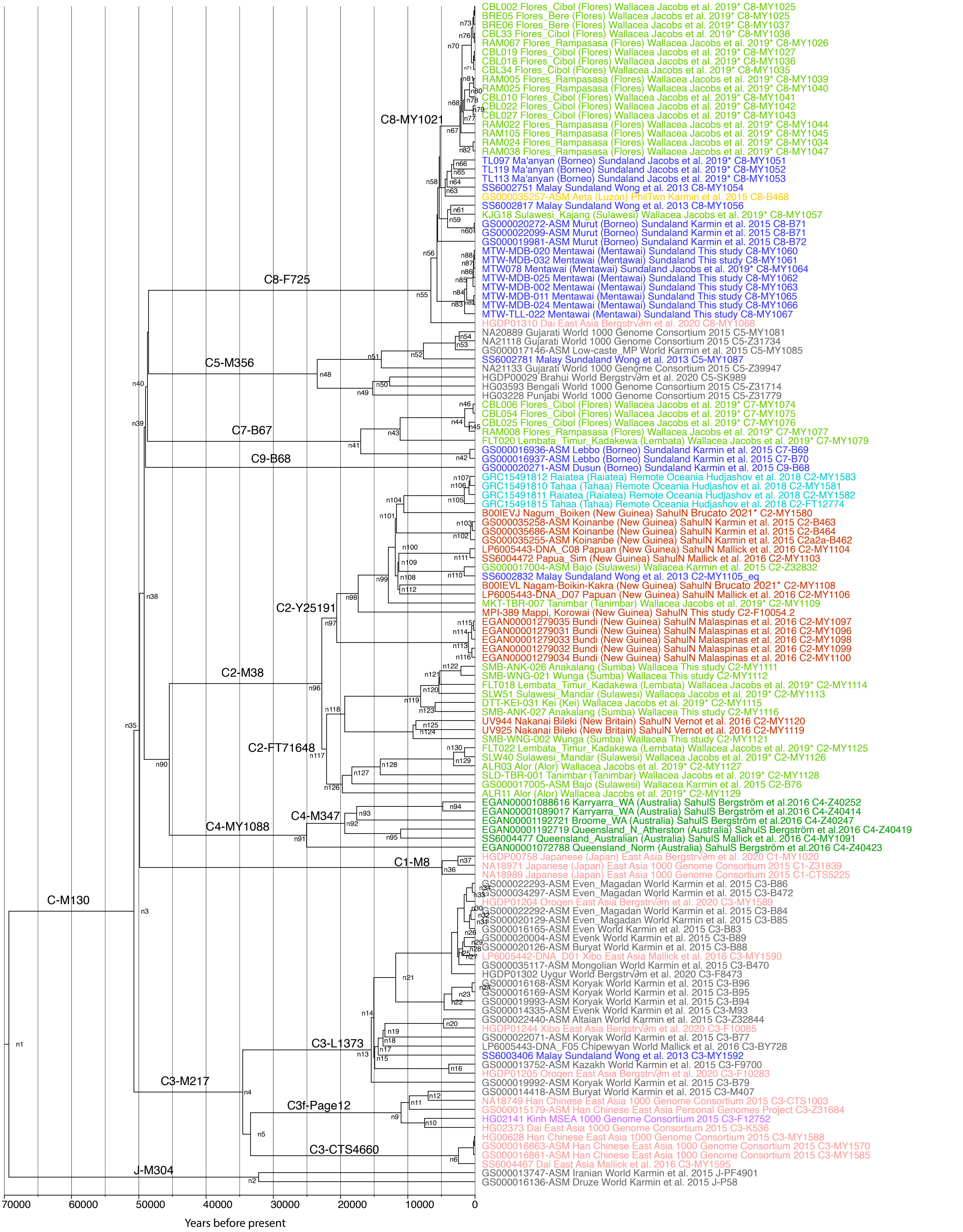

Figure S2. Dated phylogenetic tree of haplogroup (hg) C-M130. Main hg labels shown on branches, tree file and more labels in File S1, node ages with CI are in Table S3. Samples from hg J-M304 are used as outgroup and calibration point is hg C-M130 (Table S2). Labels colorcoded as on Figure 1.

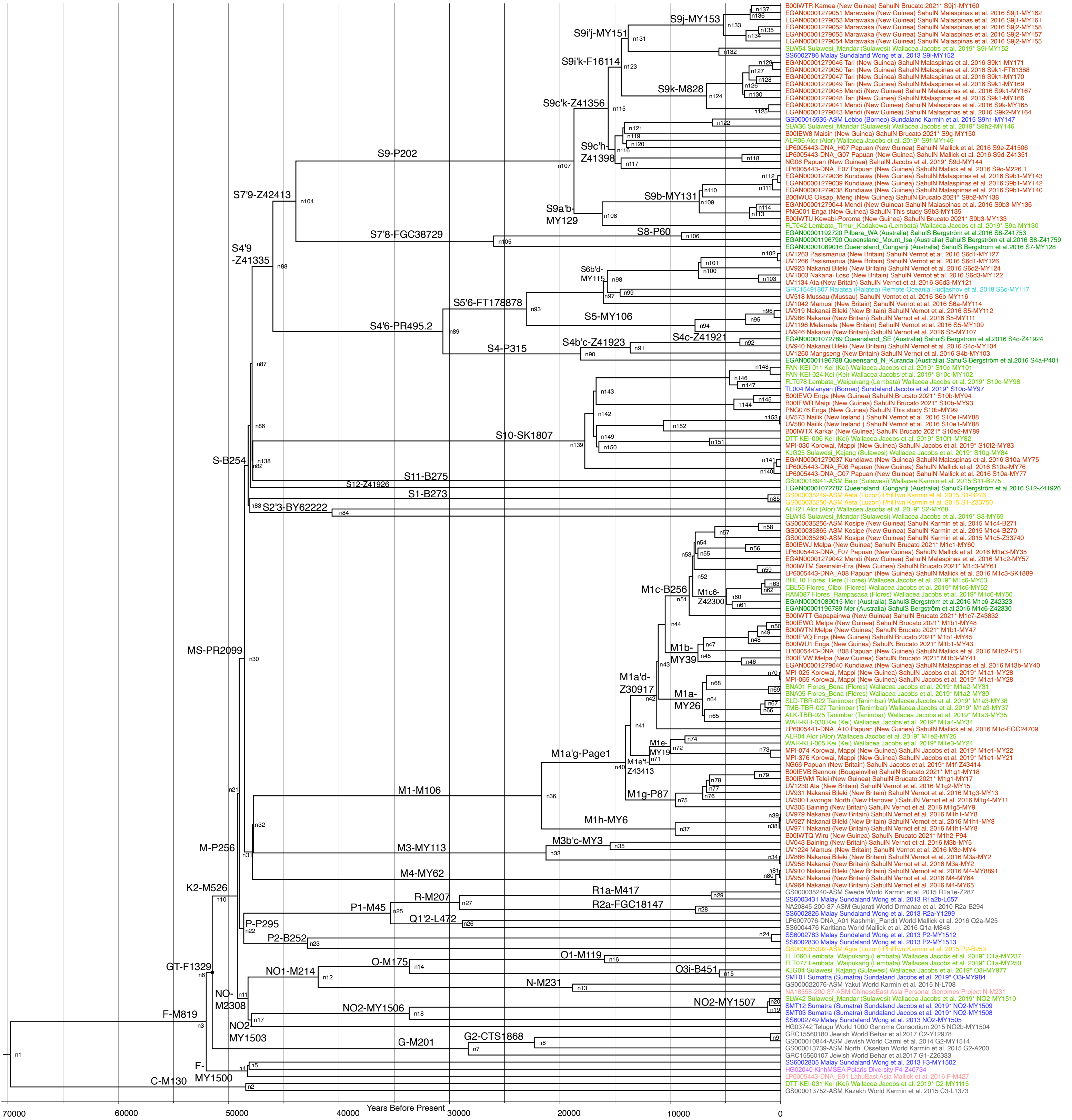

Figure S3. Dated phylogenetic tree of haplogroup (hg) MS-PR2099. Main hg labels shown on branches, tree-file with more labels in File S2, node ages with CI are in Table S4. For phylogeneitc structure, similarly for the hg O-M175 tree (Figure S4, File S3), same set of samples from hgs N-M231, NO2-MY1503, P-P259, G-M201, F-MY1500 are included, samples from hg C-M130 are used as outgroup and calibration point is GT-F1329 (Table S2). Labels colorcoded as on Figure 1.

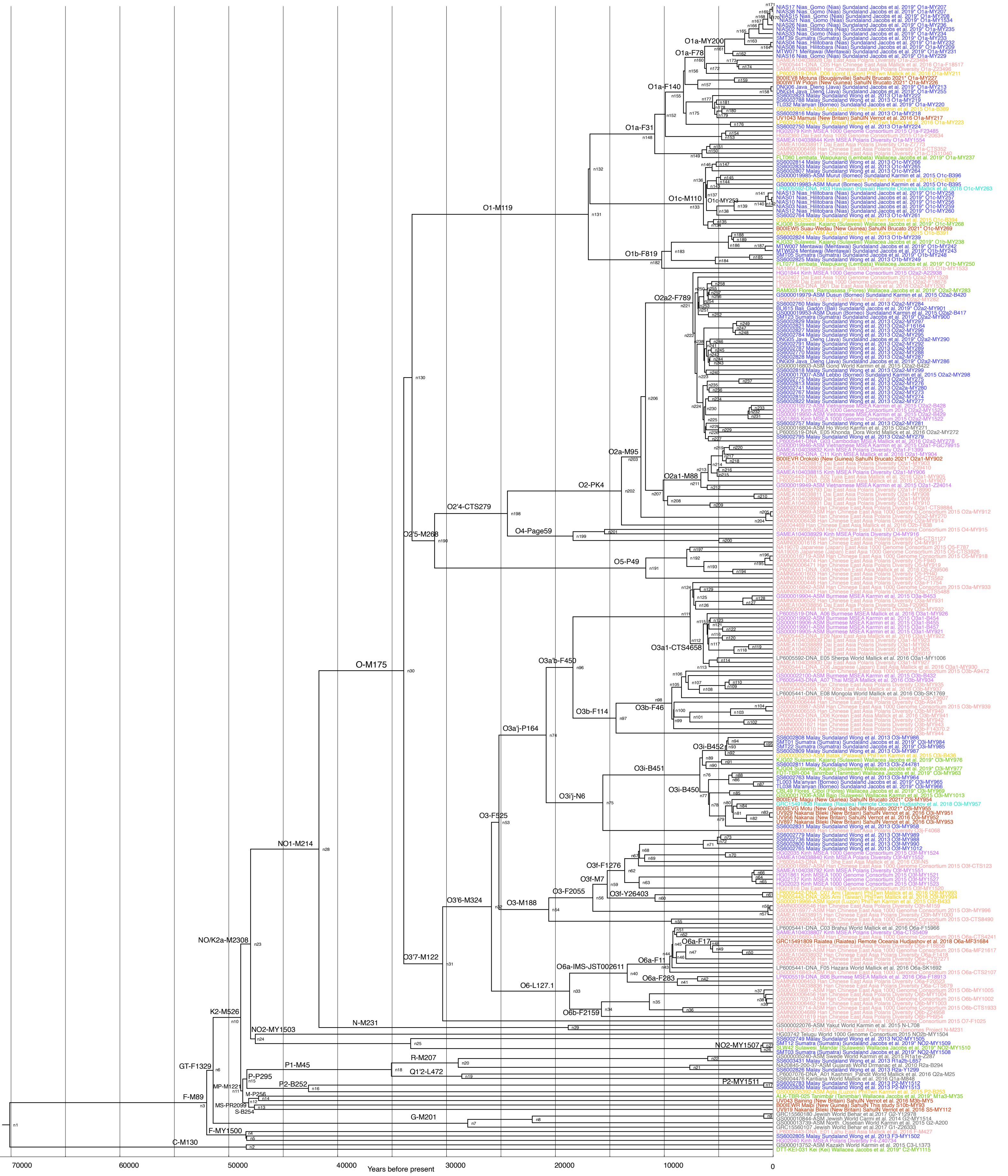

Figure S4. Dated phylogenetic tree of haplogroup (hg) O-M175. Main hg labels shown on branches, tree file and more labels in File S3, node ages with CI are in Table S5. For phylogeneitc structure, similarly for the hg MS-PR2099 tree (Figure S3, File S2), same set of samples from hgs N-M231, NO2-MY1503, P-P259, G-M201, F-MY1500 are included, samples from hg C-M130 are used as outgroup and calibration point is GT-F1329 (Table S2). Labels colorcoded as on Figure 1.
